# A Stacked Back Side-Illuminated Voltage Domain Global Shutter CMOS Image Sensor with a 4.0 μm Multiple Gain Readout Pixel †

**DOI:** 10.3390/s20020486

**Published:** 2020-01-15

**Authors:** Ken Miyauchi, Kazuya Mori, Toshinori Otaka, Toshiyuki Isozaki, Naoto Yasuda, Alex Tsai, Yusuke Sawai, Hideki Owada, Isao Takayanagi, Junichi Nakamura

**Affiliations:** 1 Brillnics Japan Inc., 6-21-12 Minami-Oi, Shinagawa-ku, Tokyo 140-0013; Japanotaka.toshinori@brillnics.com (T.O.); isozaki.toshiyuki@brillnics.com (T.I.); yasuda.naoto@brillnics.com (N.Y.); sawai.yusuke@brillnics.com (Y.S.); owada.hideki@brillnics.com (H.O.); takayanagi.isao@brillnics.com (I.T.); nakamura.junichi@brillnics.com (J.N.); 2 Brillnics Inc., Guangming 6th Rd., Zhubei City, Hsinchu County 302, Taiwan; alex.tsai@brillnics.com

**Keywords:** CMOS image sensor, voltage domain global shutter, stacked sensor, back side illumination, high dynamic range, single exposure, multiple gain readout, high full well capacity, low noise, multiple exposure

## Abstract

A backside-illuminated complementary metal-oxide-semiconductor (CMOS) image sensor with 4.0 μm voltage domain global shutter (GS) pixels has been fabricated in a 45 nm/65 nm stacked CMOS process as a proof-of-concept vehicle. The pixel components for the photon-to-voltage conversion are formed on the top substrate (the first layer). Each voltage signal from the first layer pixel is stored in the sample-and-hold capacitors on the bottom substrate (the second layer) via micro-bump interconnection to achieve a voltage domain GS function. The two sets of voltage domain storage capacitor per pixel enable a multiple gain readout to realize single exposure high dynamic range (SEHDR) in the GS operation. As a result, an 80dB SEHDR GS operation without rolling shutter distortions and motion artifacts has been achieved. Additionally, less than −140dB parasitic light sensitivity, small noise floor, high sensitivity and good angular response have been achieved.

## 1. Introduction

Global shutter (GS) CMOS image sensors (CISs) are required in many imaging applications, such as machine vision, automotive and AR/VR applications, attempting to eliminate rolling shutter (RS) distortion and the flash-band artifact, which are seen with RS pixel sensors, and in removing a mechanical shutter.

Table 1 shows a summary of approaches to realize GS CISs. Here, a use of the pinned photodiode (PD) is assumed for high-performance image sensors. In CISs, a pixel output line is shared by all the pixels in the same column. Due to this structure, in the GS pixel, an additional in-pixel analog memory is needed to store the signal until it is read out from the pixel array. Generally, there are two approaches for the in-pixel analog memory; one is to store the signal charge before the charge-to-voltage conversion takes place, which is referred to as the charge domain GS pixel [1,2,3,4,5,6], and the other is to store a voltage signal after the charge is converted to a voltage, which is referred to as the voltage domain GS pixel [7,8,9]. These two approaches can be realized by both the front-side-illuminated (FSI) devices and the back-side-illuminated (BSI) devices. One more option that is considered for the voltage domain GS pixel is a stacked BSI approach [8].

In general, the charge domain GS pixel features relatively good noise performance, since correlated double sampling (CDS) can be employed to reduce noise. However, its optical performance items, such as its achievable full well capacity (FWC), angular response (AR) and the shutter efficiency (the inverse of the parasitic light sensitivity (PLS)), are degraded due to the need to place the charge memory inside a pixel.

The performance of the voltage domain GS pixel is generally much poorer than that of the charge domain GS pixel, since it requires additional transistors and sample-and-hold (S/H) capacitors inside a pixel, which in turn results in a larger pixel size and lower optical performance when it is normalized by the pixel size. While PLS of in-pixel analog memories is smaller than the charge mode GS pixels because the signal charge is amplified by the source follower amplifier, the noise performance is limited by the kTC noise of the S/H capacitors.

To enhance the GS pixel performance, a voltage domain, stacked BSI approach has been introduced. A pixel-level interconnection [10] is needed to realize the GS operation and it is widely used in infrared focal-plane arrays, where an IR detector/pixel (not necessarily a Si detector) array is stacked with signal readout electronics built in Si integrated circuits together using a pixel-level micro-bump. The same approach was reported in pure Si devices [8]. With this approach, an extremely low PLS will be obtained, as the S/H capacitor that stores the signal voltage is fully shielded from any stray light. Furthermore, a conventional BSI pixel performance is obtained, because the upper pixel structure is basically identical to a regular BSI pixel structure. The problem of high read noise still remains even with the stacked BSI voltage domain GS pixel, but it can be alleviated due to a potentially larger signal storage capacitance and/or clever circuit technique.

A prototype CIS with 4.0 μm voltage domain GS pixels using a 45 nm/65 nm stacked CMOS process has been developed as a proof-of-concept vehicle. An additional capacitance is added in the first layer to realize single exposure high dynamic range (SEHDR) with dual conversion gains and/or multiple exposure (MEHDR), and four signal storage capacitors are implemented in the second layer lower pixel to sample four signal levels.

This paper is organized as follows; design of the new stacked BSI voltage domain GS CIS and its operation modes are described in Section 2. Measurement results and sample images are shown in Section 3, and conclusions are given in Section 4. This paper is the expanded version of our published paper [11], chip micrograph, measurement results of signal to noise ratio, fixed pattern noise, a noise estimation and some sample images are added.

## 2. New Stacked BSI Voltage Domain Global Shutter CMOS Image Sensor Design

In this section, the stacked structure of our new voltage domain GS CIS and the operation timings/modes are described, followed by estimation of temporal noise.

### 2.1. Design Concept and Key Technologies

**Figure 1:**
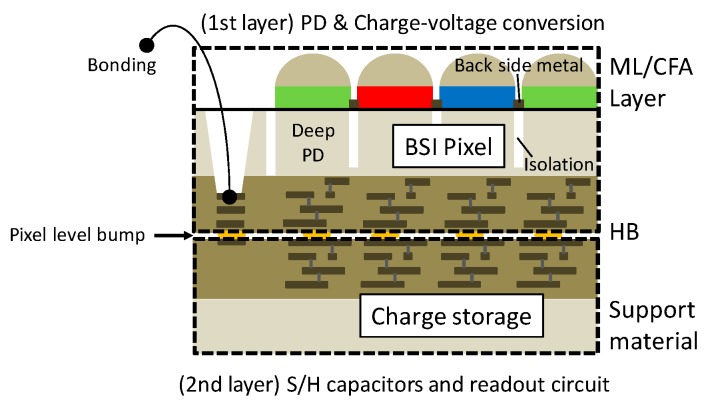
Cross sectional structure of the stacked backside-illuminated (BSI) pixel array. The micro lens (ML), color filter array (CFA) and deep photodiode (PD) are on the first layer. The sample and hold (S/H) capacitors and readout circuit are on the second layer and these two layers are connected by pixel level hybrid bump (HB).

Figure 1 shows a cross sectional structure of the stacked pixel array. The stacked pixel structure consists of the PD and charge-voltage conversion region (first layer) and the charge storage region (second layer). The charge storage region is formed on the fully light-shielded bottom stacked layer. On the other hand, BSI PDs are located in the top layer. Thus, the PD can be extended as far as possible on the top layer. This configuration offers an extremely small PLS, a high saturation signal and high optical performance of PD compared to the charge domain of the GS pixels, where in-pixel memory is located inside a pixel. Furthermore, the capacitance of in-pixel memories can be designed to be larger on the second layer, which reduces reset noise (kTC noise) of these memories.

**Figure 2:**
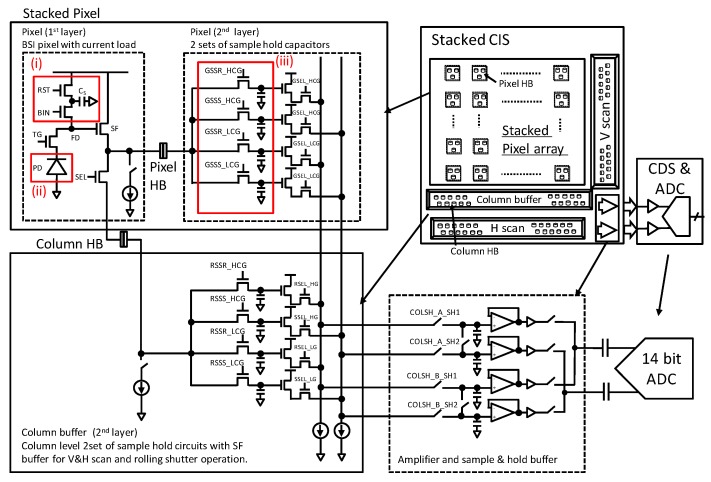
Schematic diagram and an off-chip signal processing block.

Figure 2 shows a schematic diagram of the stacked GS CIS and an off-chip signal processing block. The top BSI pixel consists of a PD, charge transfer gate (TG), floating diffusion (FD), source follower amplifier (SF), reset switch (RST), row select switch (SEL), current source and additional binning transistor (BIN) and capacitor (Cs). The charge storage region and the other driving circuits are located in the bottom layer. This SEHDR GS pixel was designed with three key technologies: (i) a multiple gain readout structure with additional BIN [12,13] and in-pixel capacitor; (ii) a high charge density PD [14]; (iii) the two sets of S/H capacitors for CDS operation in both high conversion gain (HCG) mode and low conversion gain (LCG) mode. The stacked pixel configuration with the two sets of different conversion gain signal storage realizes the GS operation with SE and ME HDR functions [9,13,14]. Furthermore, in order to increase the photo-electron conversion gain (C.G.) for low noise, a non-shared FD structure, which has a small FD capacitance owing to the small gate overlap capacitance of TG, is designed. Also, the additional SEL transistor enables SEHDR rolling shutter (RS) operation with lower noise.

The analog-to-digital conversion is carried out via an off-chip analog front end (AFE) with CDS. The variation of the offset voltage between the HCG/LCG-RST readout path and the HCG/LCG-SIG readout path including the SFs and amplifiers located in the second layer can be removed by a fixed pattern noise (FPN) cancellation operation, using the output signals of the output amplifiers with pixel output signals or the amplifiers inputs shorted as a reference.

**Figure 3:**
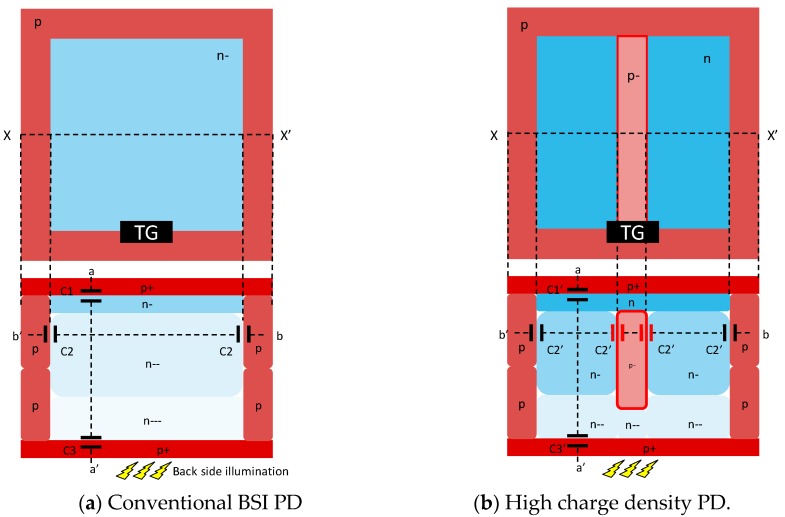
Top view and a cross-sectional view along *X*-*X*’ of a conventional backside-illuminated (BSI) photodiode (PD) and the high charge density PD [14]. TG is the transfer gate.

Figure 3 shows the top and cross-sectional views of the conventional BSI PD and our high charge density PD [14] which is adopted for this stacked GS CIS. In order to increase the boundaries of the PN junction for larger PD capacitance, a buried p-layer inside of the n-layer of the PD is formed. The buried p-layer is connected to the ground through the p-well isolation. This PD structure allows PD pinning potential to be lower than that of the conventional PD structure even though FWC is increased by the additional n-type photodiode dosage. Compared to the conventional PD structure, two times higher or more FWC is achieved with this PD structure.

### 2.2. Operation Modes

**Figure 4:**
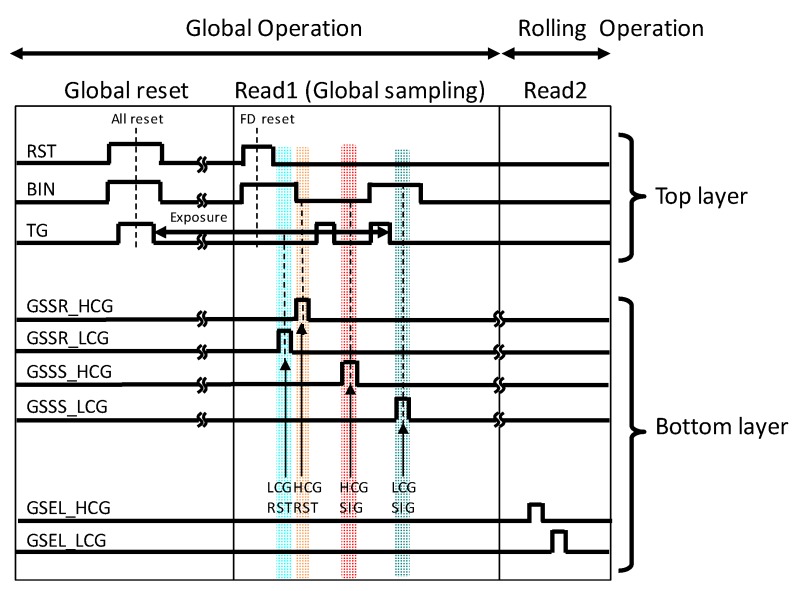
Timing sequence of the global shutter (GS) multiple gain readout operation.

Figure 4 shows a timing sequence of the GS operation, where the shutter release (Global reset), the global HDR sampling (Read1) and data readout from the pixel S/H capacitors (Read2) are performed. For the GS operation, first, all the PDs, FDs and C_S_s are reset at the same time (Global reset). After the exposure, four signals (HCG-RST, HCG-SIG, LCG-RST and LCG-SIG) are stored in the S/H capacitors in the bottom layer (Global sampling). More specifically, the LCG-RST and HCG-RST levels are stored in the S/H capacitors and then the photoelectrons in the PD are transferred to FD (first transfer) and the HCG-SIG level is stored with FD separated by BIN. At last, BIN turns ON and the LCG-SIG level is stored after the second charge transfer. This sequence enables the SEHDR operation. Then, all the signals stored in the S/H capacitors are fed to the off-chip ADC and memories row by row (Read2). To remove the kTC noise generated at reset of C_FD_, the CDS operation for both HCG and LCG signals will be realized by subtracting HCG-RST from HCG-SIG, and LCG-RST from LCG-SIG under the conditions of small kTC noise of S/H capacitors. Because the sense node capacitance at LCG (i.e., C_FD_ + C_S_) is designed larger than the PD FWC, high sensitivity by minimized C_FD_ and high FWC by larger C_PD_ in a single exposure can be realized by this operation. In other word, SEHDR with GS operation will be achieved. 

**Figure 5:**
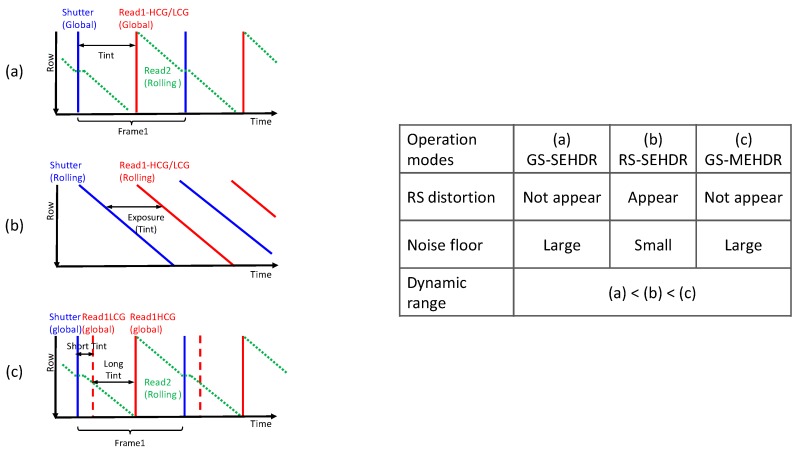
Shutter and readout timings for GS-SEHDR (**a**) RS-SEHDR; (**b**) and GS-MEHDR (**c**) mode.

The shutter and readout timings for GS and RS modes, both operating in the SEHDR mode, are shown in Figure 5a,b, respectively. In the GS mode, RS distortion does not appear because HCG and LCG signals are stored in the in-pixel S/H capacitors until they are read out from the pixel array. Owing to this operation, SEHDR with GS operation will be achieved. On the other hand, in the RS mode, these signals are stored in column S/H capacitors, of which capacitance is much larger than the in-pixel S/H capacitance and a CDS will be performed. Thus, low noise, SEHDR will be realized. However, RS distortion appears in the RS mode.

Further DR enhancement is attempted with the ME scheme [9]. In the GS readout sequence in Figure 5c, low and high conversion readouts are carried out for short exposure signals and long exposure signals, respectively, to perform an HDR operation. In this operation, longer exposure with high gain covers low light portions while shorter exposure with low gain covers high light portions of the scene. With this operation, an over 100dB MEHDR with a quasi GS operation will be realized. However, the motion artifact appears because of the ME operation, though it should be less noticeable compared to the RS motion artifact.

### 2.3. Noise Estimation

As shown in Table 1, the biggest disadvantage of the voltage mode GS CISs is the larger read noise. The primary noise sources are the kTC noise and the SF noise, associated with the signal sampling at the sampling capacitor *C_S/H_*. In this section, an estimation of these noise components is described.

The input referred kTC noise of S/H capacitors after CDS is given by

(1) $$n=\frac{1}{A_{V}\cdot C.G.}\cdot \sqrt{\frac{2kT}{C_{S/H}}}$$

With the target *C.G.* of 190 μV/e^−^, (1) yields 2.65 e^−^.

The input-referred noise of the SF is given by

(2) $$\begin{array}{ll} v_{n\_sf}^{2}\left(f\right) & =\left(i_{n_{out_{SF}}}^{2}+i_{n_{out_{LD}}}^{2}\right)/g_{m_{SF}}^{2}\\ & =\frac{8}{3}\cdot \frac{kT}{g_{m_{SF}}}\cdot \left(1+\frac{g_{m_{LD}}}{g_{m_{SF}}}\right) \end{array}$$

where *g_mSF_*, *g_mLD_*, *k* and *T* denote the transconductance of the SF transistor, the transconductance of the load transistor, the Boltzmann constant and the absolute temperature, respectively. The resulting noise voltage is calculated by integrating (2) over the SF’s noise bandwidth (BW) of

(3) $$BW=\frac{\pi }{2}\cdot \frac{g_{m\_SF}}{2\pi C_{L}}$$

where *C_L_* is the load capacitance of the source follower that corresponds to the sampling capacitor *C_S/H_* in Figure 2, and is given by

(4) $$v_{n\_SF}^{2}=\frac{2}{3}\cdot \frac{kT\cdot }{C_{S/H}}\cdot \left(1+\frac{g_{m\_LD}}{g_{m\_SF}}\right)\;=\frac{2}{3}\cdot \frac{kT}{C_{S/H}}\cdot \left(1+\sqrt{\frac{{\left(W/L\right)}_{LD}}{{(W/L)}_{SF}}}\right)$$

With designed parameter values, (4) yields 1.88 e^−^. After CDS, the resulting SF noise is estimated to be 2.65 e^−^.

Therefore, it is estimated that the total temporal noise after CDS is:

(5) $$n=\sqrt{{\left(2.65\right)}^{2}+{\left(2.65\right)}^{2}}=3.8e^{-}$$

## 3. Characterization Results

In this section, several characterization results and sample images captured by the fabricated chip are described.

### 3.1. Fabrication and Characterization Results

**Figure 6:**
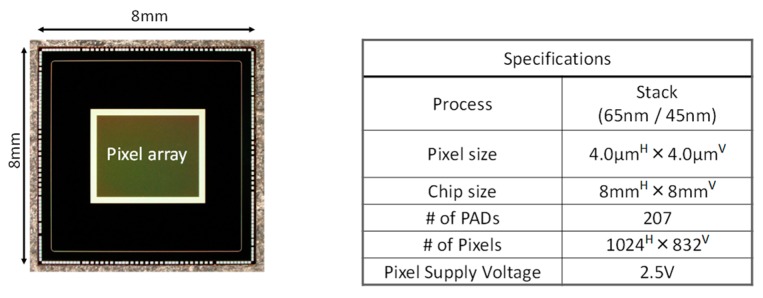
Chip micrograph and specifications of our stacked back side-illuminated voltage domain global shutter complementary metal-oxide-semiconductor (CMOS) image sensor.

Figure 6 shows a chip micrograph and specifications of the chip that was fabricated in 65 nm/45 nm stacked BSI CIS process [11]. Even with the low pixel supply voltage, the high FWC is realized because of high charge density PD and multiple gain readout structure with additional BIN transistor.

**Figure 7:**
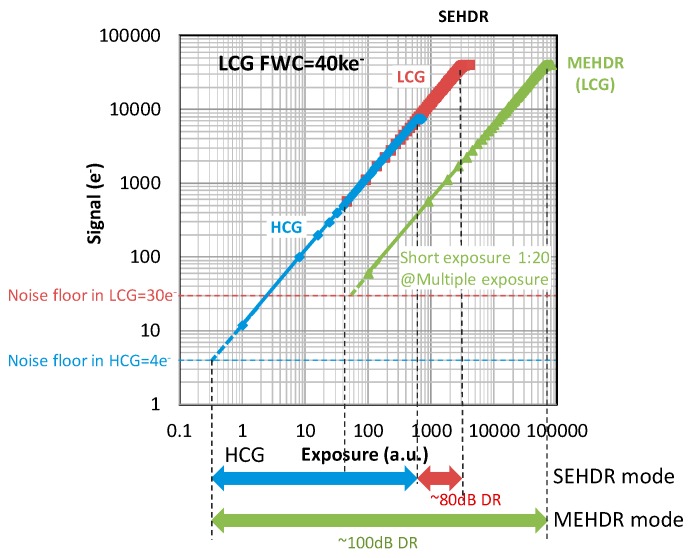
Photo conversion characteristics of high conversion gain (HCG) mode and low conversion gain (LCG) mode. The signal electrons were measured by changing exposure time under a constant light condition while different illuminance levels were set for the single exposure high dynamic range (SEHDR) and multiple exposure high dynamic range (MEHDR) measurements. Readout noise equivalent electrons in the HCG mode and the LCG mode were measured under the dark condition at the integration time of 300 us.

A photo-electron conversion plot in the single and multiple exposure GS operation is shown in Figure 7. A full well capacity of 40 ke^−^ has been obtained with the high charge density PD [14] and multiple gain readout operation. Furthermore, with the non-shared FD structure, 190 μV/e^−^ high conversion gain has been achieved, which in turn results in low read noise of 4 e^−^. This value agrees well with the estimated value shown in Section 2.3. Therefore, the root cause of the read noise is the kTC noise and SF noise. 80 dB SEHDR GS operation has thus been achieved with multiple gain readout and two sets of S/H capacitors.

Also, as shown in Figure 7, employing a dual exposure, dual C.G. scheme, maximum linear light response range is boosted 160 times with a 1:20 exposure ratio, compared to the HCG single exposure case, which results in DR of 102 dB.

**Figure 8:**
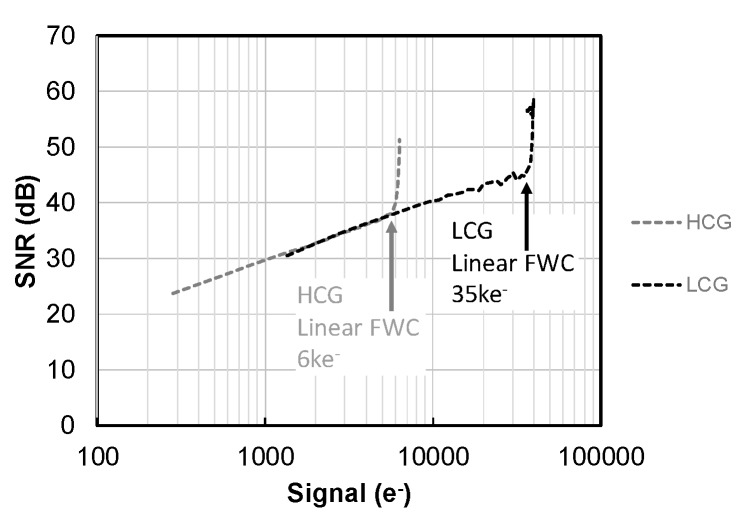
Signal to noise ratio (SNR) of high conversion gain (HCG) mode and low conversion gain (LCG) mode under the conditions of constant object illuminance and average of 100 frames.

Figure 8 shows the signal to noise ratio (SNR) of HCG and LCG mode. Linear FWC of HCG is 6 ke^−^ and LCG is 35 ke^−^, respectively. With the conjunction point being set at near the saturation level of HCG, no degradation of the SNR between HCG and LCG is observed.

**Figure 9:**
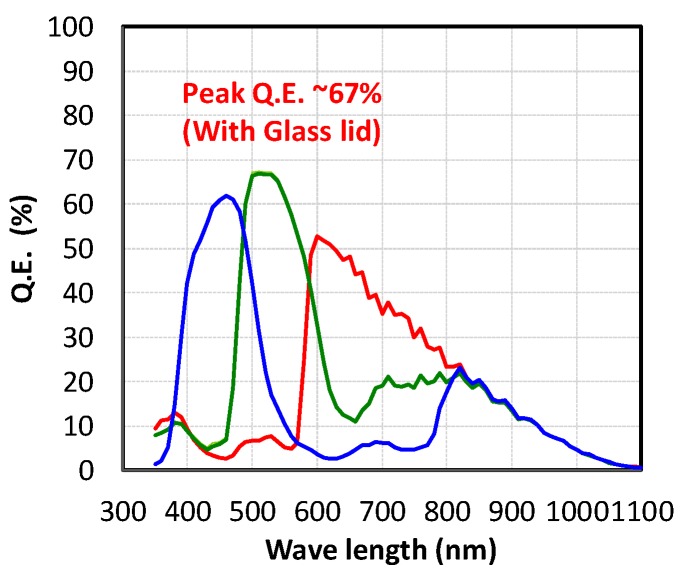
Quantum efficiency (Q.E.).

**Figure 10:**
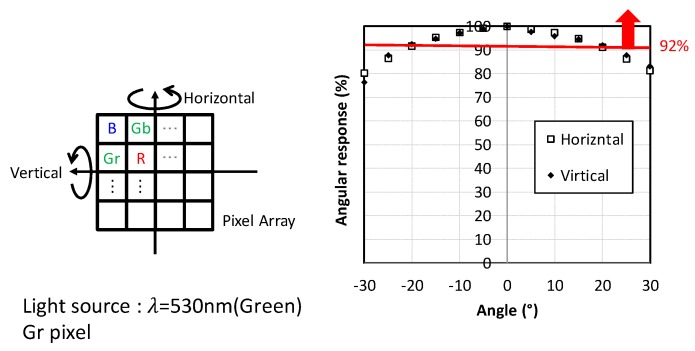
Angular response (AR) of Gr pixels. A 530 nm light source is used and the output levels of measured pixels are around one half the saturation level.

Figure 9 shows a quantum efficiency (Q.E.) plot and Figure 10 shows angular response (AR) of green pixels in the Bayer configuration, respectively. Although microlens structure has not been optimized for this pixel size, 67% peak Q.E. with glass lid and 92% AR at ±20° have been achieved. In addition, AR for both horizontal and vertical directions exhibit no significant difference. The key factor of achieving these high optical performances of this GS CIS is the adoption of the stacked BSI pixel technology.

**Figure 11:**
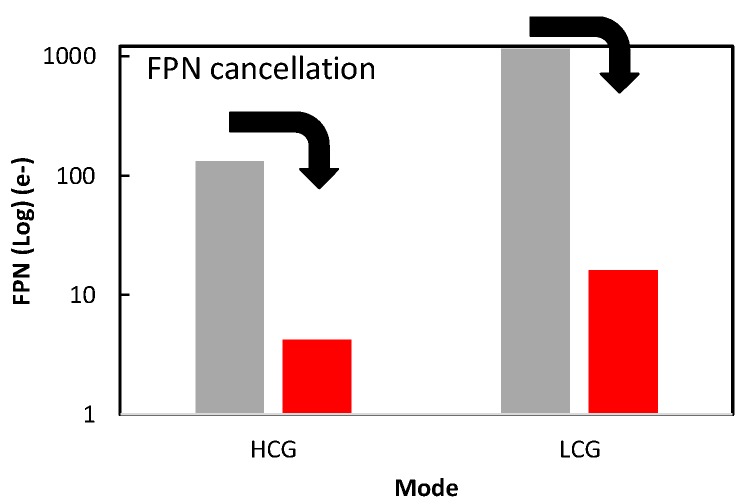
Measured fixed pattern noise (FPN) of high conversion gain (HCG) mode and low conversion gain (LCG) mode, with and without FPN cancellation operation under the conditions of 300 µs exposure and average of 100 frames.

Figure 11 shows measurement results of FPN with/without FPN suppression. With this FPN cancellation operation, the FPN caused by two sets of readout paths including SF transistor variation has been sufficiently removed. The remaining FPN sources are considered charge injection and clock feedthrough of S/H switches which cannot be removed by this FPN cancellation operation, and suppression of these FPN components is our future work.

### 3.2. Sample Images

**Figure 12:**
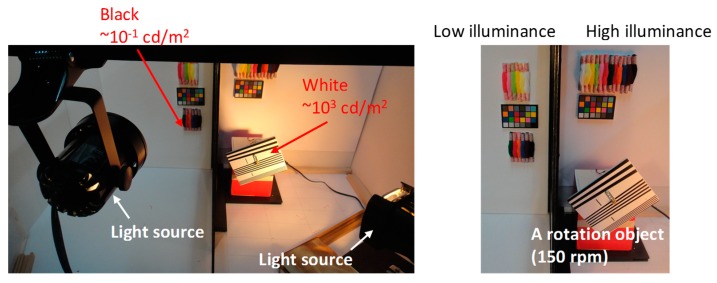
Imaging environment.

Figure 12 shows the imaging environment for confirmation of SEHDR and GS operations. To confirm the SEHDR operation, several color objects are placed in two boxes, illuminated by a high light and a low light, respectively. This setting creates a >80 dB difference in object illuminance. Additionally, a 150-rpm rotating object with straight lines is put in the highly illuminated box for confirmation of the RS distortion.

**Figure 13:**
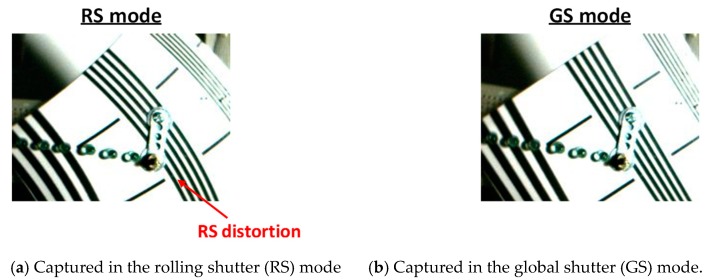
Sample images captured by rolling shutter (RS) and global shutter (GS) modes.

Figure 13a,b shows the sample images captured in the GS and RS modes with 15 frames per seconds. The lines in the resolution test pattern are straight, however the reproduced lines are curved in RS mode due to the RS distortion. On the other hand, in GS mode, the reproduced lines are straight.

**Figure 14:**
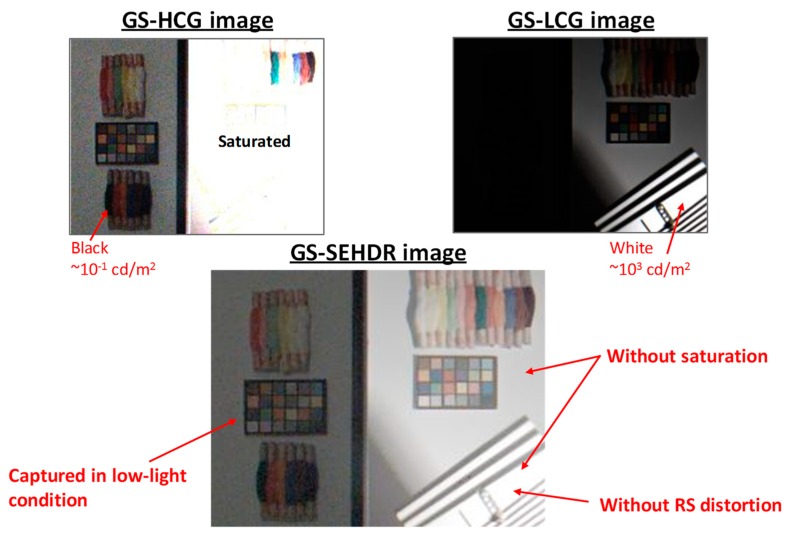
Sample images captured in the global shutter (GS) single exposure high dynamic range (SEHDR) mode with 15 frames per second (fps).

Figure 14 shows HDR GS images captured in the GS mode. Despite the 80 dB difference between the two boxes, all the color objects which are illuminated by high light and low light are captured because of the SEHDR operation. In addition, the rotating object is successfully captured without RS distortion.

**Figure 15:**
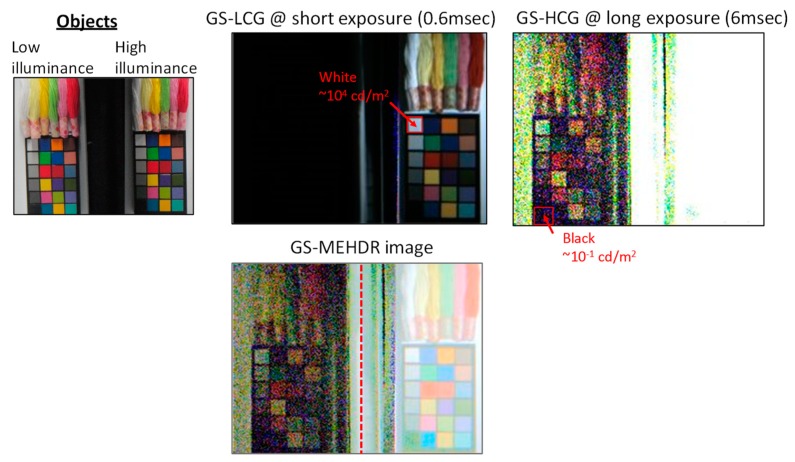
Sample images captured in the global shutter (GS) multiple exposure high dynamic range (MEHDR) mode.

Figure 15 shows another HDR image captured in the GS, MEHDR mode, under around 100 dB difference condition. Even with the 100 dB difference in the illumination levels, all the color objects are clearly captured without saturation.

## 4. Conclusions

A voltage domain GS CIS with SEHDR 4.0 μm pixels has been developed using the stacked BSI technology. Table 2 summarizes the sensor performance. In the stacked voltage domain GS CIS, the large size S/H capacitors are laid out in the bottom layer, which has achieved less than −140 dB PLS and smaller noise floor of 4e^−^. In addition, high optical performance of 74% peak Q.E. and more than 90% AR at ±20° have been achieved with BSI technology for the top layer. Also, 80 dB SEHDR GS operation has been achieved with a high charge density PD, multiple gain readout with two sets of S/H capacitors. Two sets of S/H capacitors enable further DR extension up to 100 dB with a ME readout operation.

Table 3 shows a comparison of GS pixel performance in single exposure systems. Compared with several-charge domain GS CISs, higher Q.E., better AR and lower PLS have been obtained with the voltage domain stacked BSI structure. Furthermore, compared with conventional voltage domain GS CISs, significant improvement of the noise floor, higher FWC and higher DR have been obtained with the multiple gain readout and high charge density PD technologies. Obtained key sensor performance will be improved further, especially in SNR and DR, and pixel size will be shrunk using minor design modifications.

## Figures and Tables

**Table 1:** Global shutter CMOS image sensors.

Mode	Charge Domain		Voltage Domain		
Process	FSI	BSI	FSI	BSI	Stack
Pixel structure	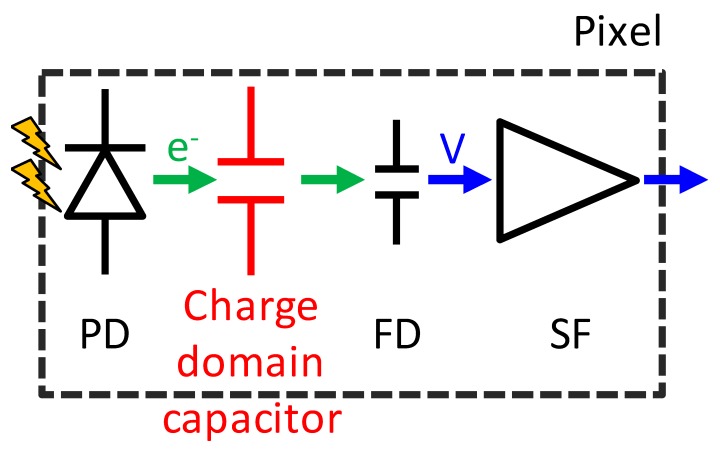		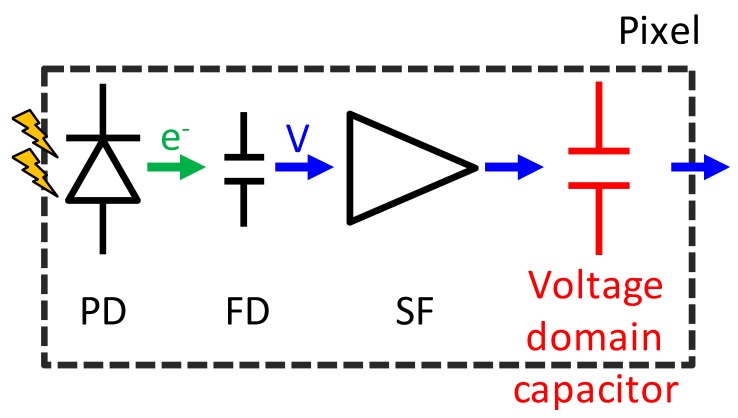		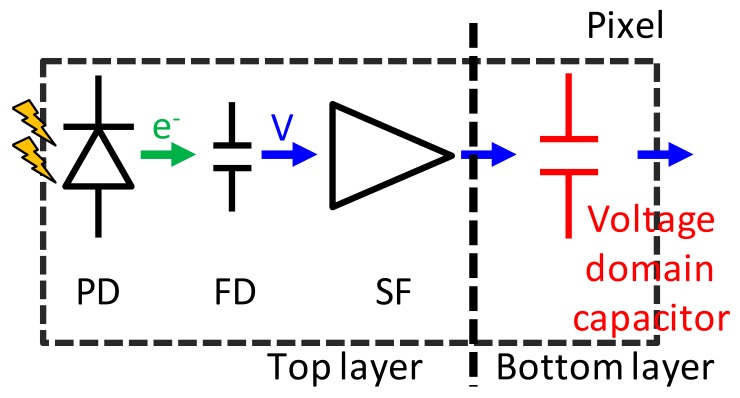
Parasitic Light Sensitivity (PLS)	Good	Poor	Good		Excellent
Read noise	Excellent		Poor		Good
			(Smaller size of voltage domain capacitors)		(Larger size of voltage domain capacitors)
Sensitivity Optical performance	Poor	Good	Poor	Good	Excellent
Others	Light shield of charge domain capacitor is needed				

**Table 2:** Sensor performance.

Specifications		
	Low gain @GS	High gain @GS
	(@RS)	(@RS)
Dynamic range	80 dB (85 dB)	
	[102 dB@1:20 of multiple exposure]	
C.G.	21 μV/e^−^	190 μV/e^−^
Linear FWC	35 ke^−^	6 ke^-^
FWC	40 ke^−^	7 ke^−^
Noise floor	30 e^−^ (13 e^−^)	4.0 e^−^ (2.1 e^−^)
Dark pixel FPN	16 e^−^ (3 e^−^)	4.2 e^−^ (1.2 e^−^)
Image lag	<1.0 e^−^	
Responsivity (5100K, CM500)	37 ke^−^/lx-s	
Peak Q.E. (Green)	74%	
AR @ ± 20°	92%	
PLS	<−140 dB	
PRNU @ 50% of FWC	0.50%	

**Table 3:** Specifications and performance comparison.

Mode	Voltage Domain	Charge Domain				Voltage Domain		Rolling Shutter
**Specification**	**This work [11]**	**IEDM2018**	**IEDM2018**	**IEDM2018**	**ISSCC2017**	**IEEE2017**	**ITE. 2016**	**IISW2017 Sensors**
	(GS mode)	Y. Kumagai et.al [4]	A. Tournier et.al [3]	T. Yokoyama et.al [5]	M. Kobayashi et.al [6]	L. Stark et.al [9]	T. Kondo et.al [8]	I. Takayanagi et.al [13,14]
Process	**Stack**	BSI	FSI	FSI	FSI	BSI	Stack	BSI
Pixel size (μm)	**4.0**	2.74	3.2	2.5	3.4	3.75	3.8	3.0
Pixel Supply voltage (V)	**2.5**	3.3	2.5	NA	3.3	NA	3.3	2.8
Peak Q.E. (Green) (%)	**74**	NA	72.9	67	NA	62.5	NA	77
AR (%) @ ± 20°	**>90**	NA	NA	50	40	NA	NA	>90
PLS (dB)	**<−140**	−80	NA	−81.6	−89	−82.5	−180	NA
Dynamic range (dB)	**80**	75	68	NA	79	60	60.5	91
Noise floor (e^−^ rms)	**4**	1.85	2	N/A	1.8	8.6	33	1.1
FWC (ke^−^)	**40**	10	16.6	6.3	16	8.5	35	45
(ke^−^/μm^2^)	**(2.5)**	(1.3)	(1.6)	@LFWC	(1.4)	(0.6)	(2.4)	(5.0)
